# Cattle movements and trypanosomes: restocking efforts and the spread of *Trypanosoma brucei rhodesiense* sleeping sickness in post-conflict Uganda

**DOI:** 10.1186/1756-3305-6-281

**Published:** 2013-09-27

**Authors:** Richard Selby, Kevin Bardosh, Kim Picozzi, Charles Waiswa, Susan C Welburn

**Affiliations:** 1 Division of Pathway Medicine and Centre for Infectious Diseases, School of Biomedical Sciences, College of Medicine and Veterinary Medicine, The University of Edinburgh, Chancellor’s Building, 49 Little France Crescent, Edinburgh EH16 4SB, UK; 2 Centre of African Studies, School of Social and Political Science, College of Humanities and Social Science, The University of Edinburgh, 58 George Square, Edinburgh EH8 9LD, UK; 3 Department of Pharmacy, Clinical and Comparative Studies, School of Veterinary Medicine and Animal Resources, Makerere University, P.O Box 7062, Kampala, Uganda

**Keywords:** Sleeping sickness, Trypanocidal treatment, Cattle movements, Restocking, Conflict, Uganda

## Abstract

**Background:**

The northwards spread of acute *T. b. rhodesiense* sleeping sickness in Uganda has been linked to cattle movements associated with restocking following the end to military conflict in 2006. This study examined the number of cattle traded from *T*. *b*. *rhodesiense* endemic districts, the prevalence of the parasite in cattle being traded and the level of trypanocidal treatment at livestock markets.

**Methods:**

Between 2008 and 2009 interviews were carried out with government veterinarians from 20 districts in Uganda, 18 restocking organisations and numerous livestock traders and veterinarians. Direct observations, a review of movement permit records (2006 to 2008) and blood sampling of cattle (n = 1758) for detection of parasites were also conducted at 10 livestock markets in *T. b. rhodesiense* endemic districts.

**Results:**

Records available from 8 out of 47 identified markets showed that 39.5% (5,238/13,267) of the inter-district cattle trade between mid-2006 and mid-2008 involved movement from endemic areas to pathogen-free districts. PCR analysis showed a prevalence of 17.5% *T. brucei* s.l. (n = 307/1758 [95% CI: 15.7-19.2]) and 1.5% *T. b*. *rhodesiense* (n = 26/1758 [95% CI: 0.9-2.0]) from these same markets. In a two-year period, between late-2006 to late-2008, an estimated 72,321 to 86,785 cattle (57, 857 by 18 restocking organisations and 10,214 to 24,679 by private traders) were imported into seven pathogen-free northern districts, including districts that were endemic for *T*. *b*. *gambiense*. Between 281 and 1,302 of these cattle were likely to have carried *T*. *b*. *rhodesiense*. While governmental organisations predominantly adhered to trypanocidal treatment, most Non-Governmental Organisations (NGOs) and private traders did not. Inadequate market infrastructure, poor awareness, the need for payment for drug treatments, and the difficulty in enforcing a policy of treatment at point of sale contributed to non-compliance.

**Conclusion:**

With increasing private trade, preventing the spread of Rhodesian sleeping sickness in Uganda requires government support to ensure mandatory trypanocidal treatment at livestock markets, investment in market infrastructure and possible drug subsidy. Mapping the northern reaches of *T. b. rhodesiense* in livestock and preparation of risk assessments for cattle trading could mitigate future outbreaks.

## Background

Human African trypanosomiasis (HAT), also known as sleeping sickness, is a deadly neglected disease caused by trypanosomes (protozoan parasites) and transmitted by the tsetse fly vector. Caused by two different trypanosome sub-species (*Trypanosoma brucei gambiense* and *T*. *b*. *rhodesiense*), the two forms of sleeping sickness are separated by the Great Rift Valley and differ in epidemiology, diagnostics, treatment and control options [1]. Both cause a significant human health burden in endemic foci among poor, subsistence-level farmers where the disease is largely underreported [2-5]. The majority of cases are due to infection with the chronic form of the disease, caused by *T*. *b*. *gambiense*, found throughout western and central Africa and mainly transmitted by human-tsetse contact [6]. Acute HAT is a zoonotic disease caused by *T*. *b*. *rhodesiense* and present throughout southern and eastern Africa where a wide range of wild and domestic animals act as reservoirs of infection [7,8]. Sleeping sickness epidemiology is driven by a variety of factors, including the nature of the parasite, tsetse flies, reservoir hosts, human populations and biophysical phenomena whose complex interactions drive the emergence of epidemics. An important force helping to structure epidemiological shifts have been social and political conflict [9-16].

Uganda is the only country known to have both Rhodesian and Gambian forms of sleeping sickness [5,17]. From 2000 to 2009, the country reported 3,775 cases of *T*. *b*. *gambiense* (out of a total of 170, 486 reported from 14 western and central African countries) and 2,848 cases of *T*. *b*. *rhodesiense* (from a total of 5,086 cases from 7 eastern and southern African countries) [18]. Both sub-species have historically remained geographically isolated with the Gambian form endemic to the West Nile sub-region in the northwest of the country and the Rhodesian form in the south-eastern Busoga region along Lake Victoria [5,6]. Any overlap between the two sub-species of human infective parasite would severely complicate effective medical surveillance, treatment and control activities, and should be considered of major economic and public health importance [19,20].

In Uganda, outbreaks of Gambian sleeping sickness between the early 1900s and 1950s were believed to be due to population movements in the West Nile sub-region while resurgence after the 1980s primarily involved civil unrest in Uganda and Sudan [10]. A combination of factors related to the establishment of colonial British rule (the 1900 colonial hut-tax, a rinderpest epidemic, intertribal wars, a smallpox epidemic, drought and famine and the large-scale movement of cattle and people) were responsible for the largest recorded sleeping sickness epidemic (caused by *T*. *b*. *rhodesiense*) which killed over 300,000 people between 1900 and 1920 along the shores of Lake Victoria [9,15,16,21]. Similarly, epidemics from 1976 to 1983 in the Busoga sub-region and 1984 to the mid-1990s in the southeast were influenced by the economic decline during and after the Idi Amin era (1971–1979) [22-24]. While the history of sleeping sickness in Uganda has been shaped by military and civil conflict, the future spread of *T*. *b*. *rhodesiense* may be driven by the restoration of peace following decades of conflict in the northern region.

Changes in population density, land-use and wildlife in Uganda have shifted the main reservoir-host for *T*. *b*. *rhodesiense* from wildlife to cattle [5,17]. In the late 1980s, *T*. *b*. *rhodesiense* moved eastward from its traditional foci along the shores of Lake Victoria through cattle movements causing over 40,000 reported cases [22]. At the same time, heavily armed Karamojong warriors began sustained cattle raids throughout the northern and eastern regions [25,26]. The northern district of Kitgum along the Sudanese border had cattle populations reduced from 156,667 in 1986 to a mere 3,239 in 1998 [27]. A period of rebellion then began in Teso sub-region that lasted until 1994 (known as the Teso War) and included half the population assembled into hastily constructed internment camps at the height of the government’s ‘scorched earth policy’ [25,26]. In West Nile and Acholi sub-regions to the north of Teso and Lango, a variety of rebel groups, population movements and internment camps had been ongoing during the same period, including the Lord’s Resistance Army (LRA) notorious for kidnapping and use of child soldiers [28,29]. With the end of the Teso insurgency, the LRA moved south through Lango sub-region, eventually entering Soroti town (the largest urban centre in Teso sub-region) in 2003. Prior to the LRA’s retreat into Sudan in 2006, nearly 2 million people in northern Uganda (approximately 50% of the population) were residing in internally displaced persons (IDP) camps, exposed to chronic over-crowding, disease, insecurity and social problems [28,29].

Numerous churches, NGOs and government schemes have aimed to address the northern region’s extreme poverty. Many of these organisations have been involved in cattle restocking, essential to provide oxen for ploughing to improve agricultural output. In 1998, between the end of the Teso War (1994) and the period of LRA activities in the eastern region (2003–2006), an outbreak of Rhodesian sleeping sickness occurred in Soroti district, north of Lake Kyoga. Driven by cattle movements from the southeast where up to 18% of cattle were found positive for *T*. *b*. *rhodesiense*[30], a case control study showed that the parasite was introduced into Brooks Corner market and dispersed from this market to other areas over time [31]. There was a threat of a rapid and sustained movement of the parasite northward [31-33]. The Ugandan government revised its livestock movement regulations, enacting a directive from the Ministry of Agriculture that a dose of trypanocide (either diminazene aceturate or isometamidium chloride, which costs roughly US$1) be injected into each animal at point of sale to eliminate the parasite, unless the animal was destined for slaughter (due to the drug’s residual period) [34]. Government attempts to enforce trypanocidal treatment at markets failed to contain the burgeoning epidemic as the disease spread further north into three new districts. In 2006 the two forms of sleeping sickness threatened to overlap, with less than 150 km separating them [19]. The threat of the overlap of the two diseases precipitated the formation of a public-private partnership, Stamp Out Sleeping Sickness (SOS), which undertook mass trypanocidal treatment and insecticidal spraying of cattle in Amolotar, Apac, Dokolo, Kaberamaido and Lira district [35]. However, the only consistent government control measure in place at this time involved the treatment of cattle at point of sale with trypanocidal drugs [34].

This study examined the risk of spread of *T*. *b*. *rhodesiense* to post-conflict northern districts between 2006 and 2008 by examining the main livestock market sites in endemic districts supplying cattle to northern areas; investigating the level and direction of inter-district trade; assessing the prevalence of *T*. *b*. *rhodesiense* in cattle traded and the degree of trypanocidal treatment applied; and looking at the potential contribution of cattle restocking organisations and private traders in disease spread.

## Methods

This study was conducted between January 2008 and July 2009 in three phases, involving a mixture of biomedical and social research, including both quantitative and qualitative methods. The first phase involved exploring the nature of livestock markets and cattle movements in *T*. *b*. *rhodesiense* endemic areas in the northern and eastern region, the second phase established the prevalence of *T*. *b*. *rhodesiense* at these same market sites, and the third phase investigated the potential for the introduction of *T*. *b*. *rhodesiense* to pathogen-free northern districts by livestock traders and restocking organisations.

### Study area

The study comprised 20 districts in the eastern and northern regions of Uganda: 8 districts in the eastern region (Amuria, Bukedea, Iganga, Kamuli, Kumi, Pallisa, Soroti and Tororo); 12 in the northern region, including 5 districts in Lango sub-region (Amolotar, Apac, Dokolo, Kaberamaido and Lira), 4 districts in the Acholi sub-region (Amuru, Gulu, Kitgum and Pader) and 3 districts in the West Nile sub-region (Adjumani, Arua and Nebbi). Cases of Gambian sleeping sickness have been reported from five of these northern districts (Adjumani, Amuru, Arua, Gulu and Nebbi) while *T*. *b*. *rhodesiense* is endemic in 11 districts, including 7 districts in the eastern region (Bukedea, Iganga, Kamuli, Kumi, Pallisa, Soroti and Tororo) and 4 districts in the northern region (Amolotar, Dokolo, Kaberamaido and Lira) [36].

**Figure 1:**
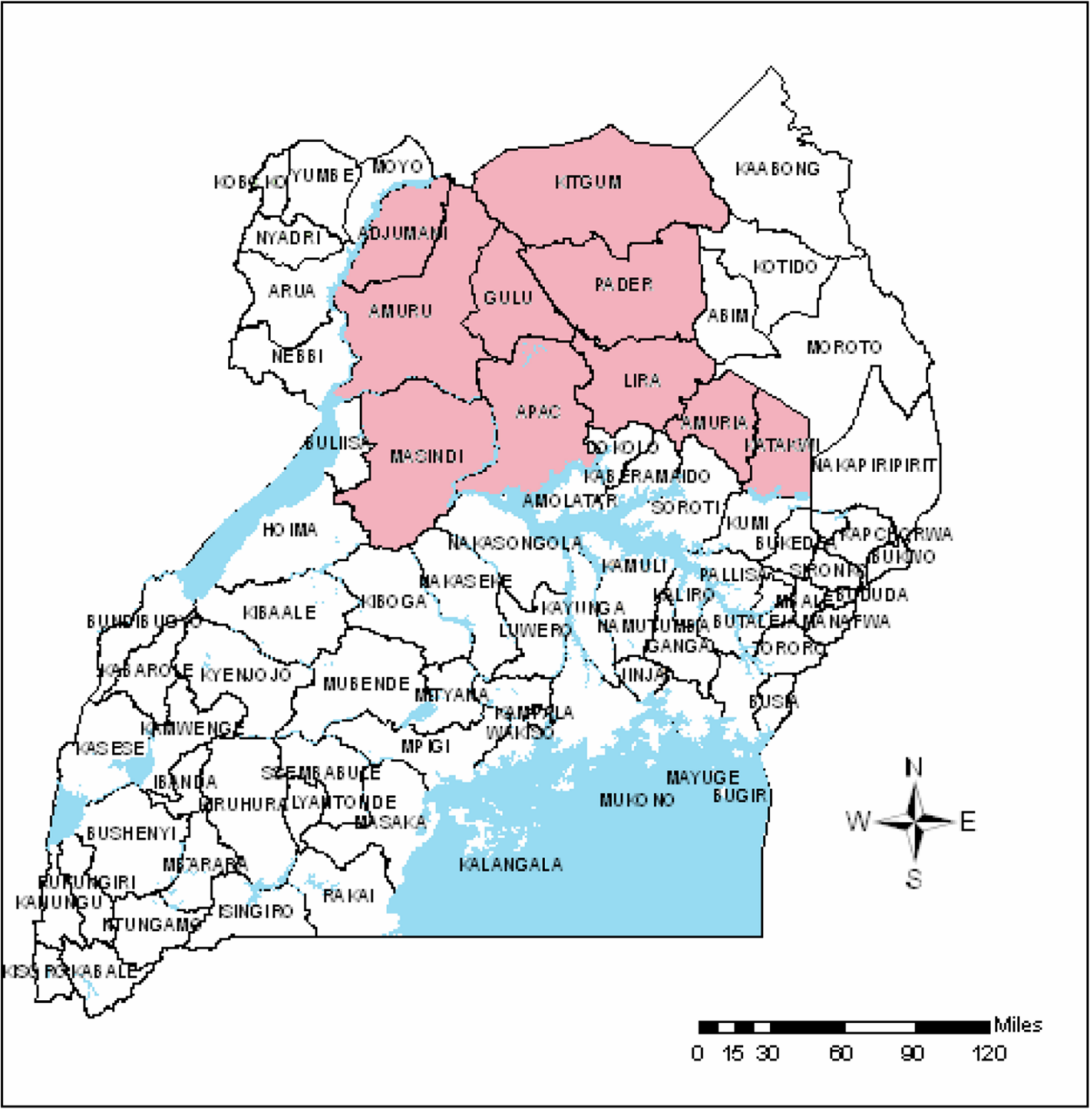
Ugandan districts with IDP camps as of 2006.

The northern and eastern regions have a human population of over 11 million. The population living below the poverty line in Uganda increases from south to north (reaching over 60% in much of northern Uganda and between 30 to 60% in the eastern region) [37]. Prior to the end of conflict in 2006, 54% of the rural population (an estimated 1,840,000 people) in Adjumani, Amuru, Apac, Gulu, Katakwi, Kitgum, Pader and Lira districts were living in IDP camps (see Figure 1) [38].

The predominately indigenous national cattle herd of Uganda was estimated at 11,434,795 million head in 2008 [39]. Approximately 2.47 million cattle were in the central region, 2.49 million in the eastern region, 3.92 million in the northern region and 2.55 million are found in the western region. Herd sizes range between 5 to 80 head of cattle and cattle trading occurs both locally and over more extensive distances with animals moved either on foot or by truck through a poorly maintained road network.

### Interviews, market records and direct observations

**Figure 2:**
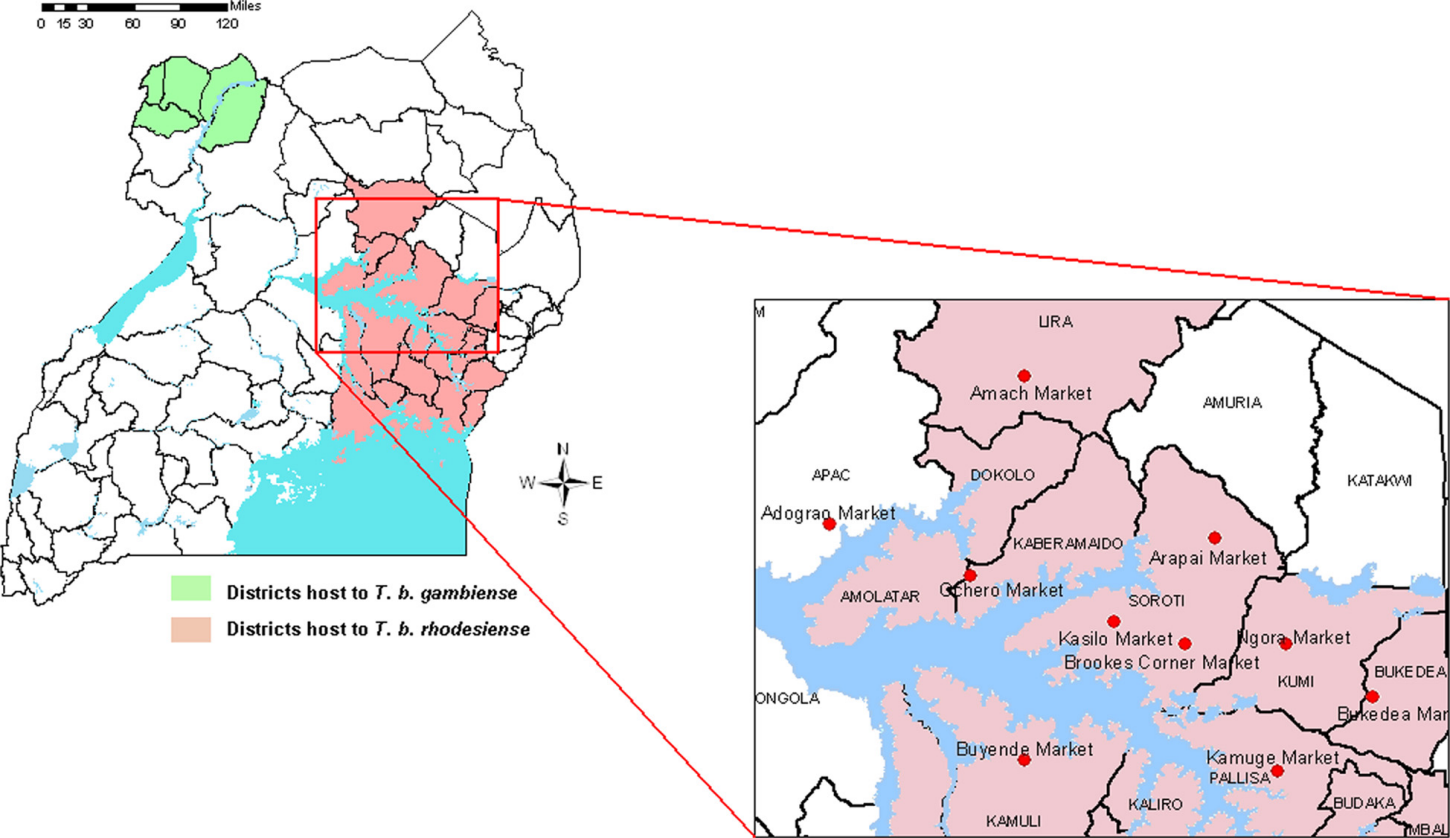
Livestock markets sampled in relation to sleeping sickness in Uganda.

In 2008 a questionnaire was carried out with the District Veterinary Officers (DVO) from 13 districts in the northern and eastern region (endemic for *T*. *b*. *rhodesiense*). Complete district-level cattle movement permit records spanning the period mid-2006 to mid-2008 were only available from Lira district. Based on information regarding the number and direction of cattle traded at markets, 10 sites perceived by DVOs as the most significant in trading cattle northward were selected for further study. These markets are shown in relation to districts known to be endemic for *T*. *b*. *rhodesiense* in 2008 (Figure 2). Each of these sites were visited at least twice over the study period. Direct observation of market infrastructure, sales practices and veterinary drug treatments (including trypanocidal treatment) were carried out and a series of semi-structured and unstructured interviews conducted with private livestock traders, farmers, NGOs, veterinarians and market staff. Official movement permit records were sought from each market for 7 different monthly periods between mid-2006 and mid-2008: June and November 2006; February, June and November 2007; and February and May 2008. The availability and quality of these records varied and analysis was undertaken on 34 full monthly records from 8 markets.

### Sample collection

Blood sampling of cattle at the 10 livestock markets was conducted during two sampling rounds between May and July 2008. Samples were randomly drawn from presenting cattle with the aim to collect 100 samples per market per visit. Sampling at market sites is generally considered to accurately reflect the prevalence of *T*. *b*. *rhodesiense* within the area surrounding the market [40]. A total of 920 samples were collected during the first round and 846 during the second for a total of n = 1766 (Table 1); 2 cards were spoiled reducing the number of samples to 1758. Blood was drawn from the ear vein of each sampled animal into two heparinised capillary tubes and applied immediately onto an FTA card (Whatman, Maidstone, Kent, UK). FTA cards were left to dry and placed together with desiccant in airtight multi-barrier pouches (Whatman, UK) prior to transport and analysis [41]. Since samples were taken early in the day, information was only available regarding the origin of each animal prior to entrance at the market site and not its future destination.

**Table 1:** Blood samples collected from 10 livestock markets

**District(market name)**	**Round 1**	**Round 2**	**Market total**
Apac (Adograo)	52	0	52
Bukedea (Bukedea)	108	110	218
Kaberamaido (Ochero)	100	28	128
Kamuli (Buyende)	100	100	200
Kumi (Ngora)	100	100	200
Lira (Amach)	100	100	200
Pallisa (Kamuge)	100	88	188
Soroti (Arapai)	60	100	160
Soroti (Brookes Corner)	100	120	220
Soroti (Kasilo)	100	100	200
Total	920	846	1,766

### Questionnaires conducted in *T*. *b*. *gambiense* endemic districts

A questionnaire was then administered in early 2009 to the DVOs of seven districts in the Acholi and West Nile sub-regions, including five districts known to be endemic for *T*. *b*. *gambiense* (Adjumani, Amuru, Arua, Gulu and Nebbi districts) and two that are not (Kitgum and Pader districts). Questions related to the extent of cattle movements, the various organisations involved and animal health inspections conducted at market sites. Official cattle movement permits were unavailable. Based on this data, an additional questionnaire was conducted with the 18 identified cattle restocking organisations active within these same districts during mid-2009. This questionnaire explored the nature of these organizations, various aspects of their operational practices and their knowledge of Rhodesian sleeping sickness.

### Data analysis

All questionnaire data was entered and analysed using Excel (Microsoft Office Excel 2007). Qualitative data was entered into Microsoft Word and analysed manually. Blood samples were analysed in two stages; firstly TBR-PCR was used to diagnose the presence of *T*. *brucei* s.l. [41,42]; samples shown to be positive for *T*. *brucei* s.l. were further analysed for the presence of *T*. *b*. *rhodesiense* by using multiplex (SRA gene) PCR [43]. The 95% confidence intervals were calculated based on the binomial distribution.

### Ethical approval

At the national and district levels, the study was conducted with the approval of the Coordinating Office for Control of Trypanosomiasis in Uganda (COCTU) as well as the District Veterinary Officers (DVOs) in each of the study districts. Verbal informed consent was also sought from each interviewed farmer, livestock trader, veterinarian and NGO.

## Results

### Livestock markets in *T*. *b*. *rhodesiense* areas

In total, 47 livestock markets were identified in the 13 districts endemic for *T*. *b*. *rhodesiense*. Market records from the 10 selected markets were divided between animals sold and moved within the district or to a different district and whether they were destined for slaughter (within a 3 week period after purchase) or breeding purposes. Due to poor record keeping, information on the intended use (slaughter or breeding) of these animals was unavailable. Standards and practice in maintaining records differed widely depending on the organisation of the DVO and market veterinarian as well as the availability of record books. Market records were not available for Adogran market in Apac district and Amach market in Lira district was excluded from the analysis since records were only available from the district-level. From the remaining 8 markets, 61% (34/56) of the full monthly records were available from the 7 different months over the study period (see Table 2). From the available data, a recorded 13,267 cattle had been sold and moved to a different district from these markets. Extrapolating to the 8 markets an estimated 73,289 cattle were traded and moved to a different district between June 2006 and May 2008, an average of 3,053 cattle each month.

**Table 2:** Available market records showing movement permits issued

**Month**	**Inter**-**district cattle movement**	**Markets with available records**	**Average cattle movement per market**	**Cattle moved at the 8 market sites per month(estimate)**
June 2006	1,150	3	383	3,064
November 2006	1,083	3	361	2,888
February 2007	1,332	4	333	2,664
June 2007	1,438	5	288	2,304
November 2007	2,296	6	383	3,064
February 2008	3,001	7	429	3,432
May 2008	2,967	6	495	3,960
**TOTAL**	**13**,**267**	**34**	**382**	**21**,**376**

Analysis of districts to where these cattle were moved showed that 39.5% (n = 5,238/13, 267) were destined for *T*. *b*. *rhodesiense*-free districts. These included the eastern districts of Katakwi (341) and Amuria (622) and the northern districts of Amuru (372), Gulu (903), Pader (1,259) and Kitgum (810) in the Acholi sub-region, Arua (308) in the West Nile sub-region, and Apac (623) in the Lango sub-region. Twelve percent (1,583/13,267) were to districts that had reported cases of *T*. *b*. *gambiense* (Amuru, Arua and Gulu). An estimated 28,949 out of 73,289 cattle (39.5%) sold at these 8 markets were moved to these seven districts between June 2006 and May 2008.

These official records underestimate the full extent of cattle movements since they were based on official movement permit records from only 8 markets of 47 identified in the 13 districts endemic for *T*. *b*. *rhodesiense* identified in 2008. In addition, during the post-conflict period many cattle traders sourced cattle directly from villages and obtained a movement permit from the DVOs office directly, evaded obtaining movement permits altogether at markets (transporting cattle illegally), or obtained a movement permit for only a few of the animals they intended to transport. These practices made accurate estimates difficult. For instance, while market-level data was not available for Amach market in Lira district (the most northern foci for *T*. *b*. *rhodesiense* at the time), between September 2007 and May 2008 a total of 11, 370 cattle were moved out of the district with permits (the only complete district-level records available from the 13 endemic districts). From these records; a total of 98.5% (n = 11,199/11,370) cattle were destined for *T*. *b*. *rhodesiense* free districts in the northern region, including Amuru (3989), Gulu (1162), Pader (2736) and Kitgum (1387) in the Acholi sub-region, Arua (130) and Adjumani (499) in the West Nile sub-region and Apac (1296) in the Lango sub-region. This included 52% (n = 5,780/11,199) moved to districts with reported cases of *T*. *b*. *gambiense* including Adjumani, Amuru, Arua and Gulu.

Market records together and interviews between 2006 – 2008 showed three important features: i) that the number of cattle traded at markets was increasing; ii) that more animals were being moved northwards of the market sites; and iii) that some animals were being traded into Sudan. Between the first (June 2006) and last (May 2008) sampling points there was an increase from an average of 383 cattle moved inter-district per market to 495 moved per market. Movement permits showed that while 65.8% of the 13,267 recorded cattle had moved north of the market district from 2006 to 2008, this had changed during this period with progressively more cattle being traded northwards. This is supported by interview data obtained from the DVOs from the Acholi and West Nile sub-regions and corresponds to the disbanding of the IDP camps after the end to conflict in late 2006. A number of interviews with DVOs and cattle traders stressed the large number of cattle illegally being transported from the eastern and northern regions across the border into Sudan, destined for slaughter in Juba. While international movement is strictly controlled and licensed, the traders were able to move animals to the border districts of Kitgum and Amuru and across the international border with relative ease.

### Prevalence of *T*. *b*. *rhodesiense* at market sites

**Figure 3:**
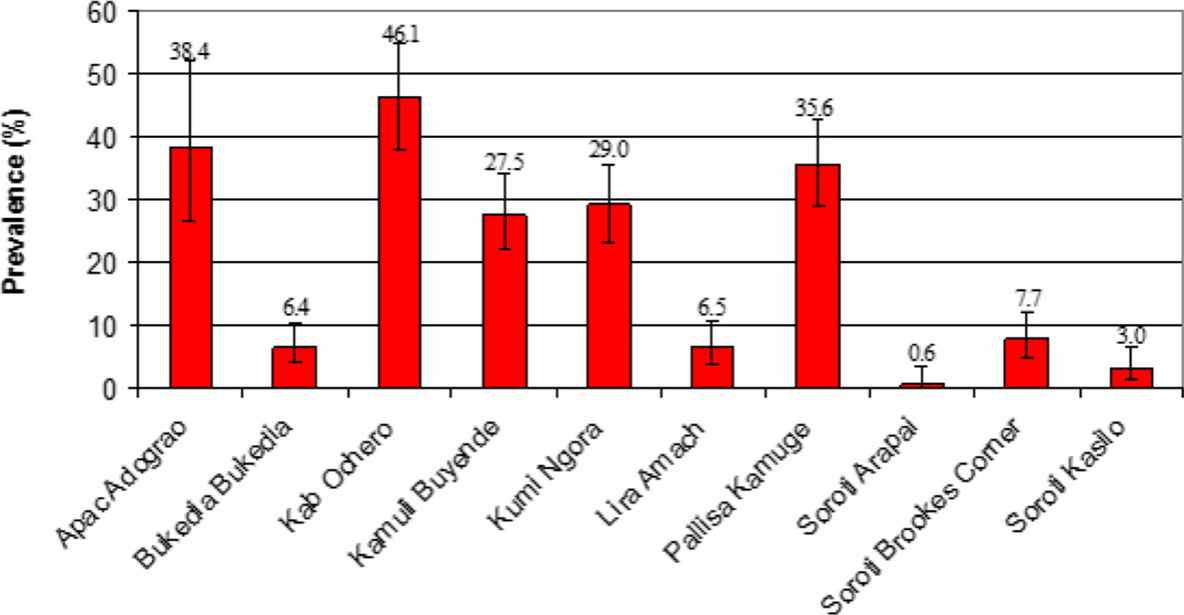
**Prevalence of *T. brucei s ***.***l. *from the 10 livestock markets sampled.**

**Figure 4:**
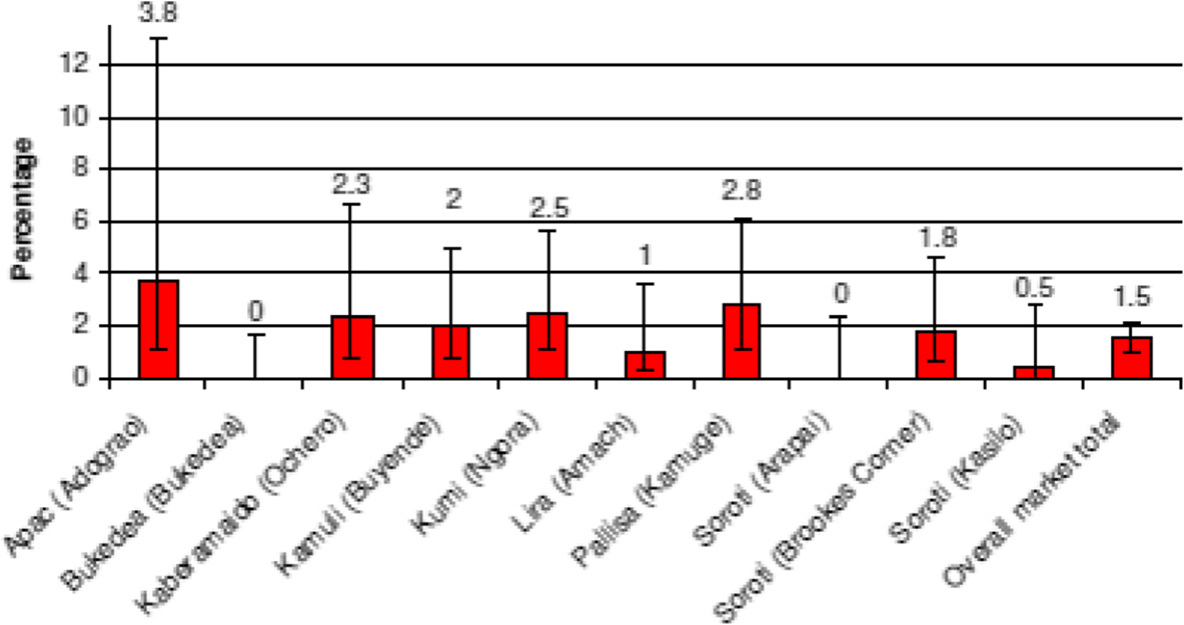
**Prevalence of *T. b. rhodesiense *in cattle sampled from the 10 livestock markets.**

PCR analysis of samples from the 10 market sites sampled showed a prevalence of 17.5% *T*. *brucei s*.*l*. (n = 307/1758 [95% CI: 15.7-19.2]) and 1.5% for *T*. *b*. *rhodesiense* (n = 26/1758 [95% CI: 0.9-2.0]) (see Figures 3 and 4). Five markets showed a prevalence of *T*. *brucei s*.*l*. in excess of 25%. Ochero market in Kaberamaido district showed a prevalence of 46.1% [95% CI: 37.4-54.7] (59/129). Five markets were trading animals with a prevalence of *T*. *brucei s*.*l* lower than 8% (the lowest being 0.6% in Arapai market [95% CI: 0–3.5]) (see Figure 4). Of the 281 cattle found to be positive for *T*. *brucei s*.*l*, 54.1% had originated from within that district while 45.9% originated from up to 19 different neighbouring districts. Animals infected with *T*. *b*. *rhodesiense* were identified in 80% of the markets (8/10). *T*. *b*. *rhodesiense* prevalence varied from 3.8% (n = 2/52 [95% CI: 0–9.1]) in Adograo market in Apac to 0% in both Arapai market (n = 0/160 [95% CI: 0–2.3]) in Soroti district and Bukedea market in Bukedea district (n = 0/218 [95% CI: 0–1.7]). The same 5 markets with the highest *T*. *brucei s*.*l*. prevalence (above 25%) were also found with the highest *T*. *b*. *rhodesiense* prevalence (above 2%) confirming the strong relationship between infection *T*. *brucei s*.*l* and *T*. *b*. *rhodesiense*. The majority of animals found positive for *T*. *b*. *rhodesiense* (81%, n = 21/26 [95% CI: 65.6 -95.9]) originated from within the market district.

Of concern is that 2 of the 26 *T*. *b*. *rhodesiense* positive cattle originated from, and were sold in, Apac district, a district that had not reported any Rhodesian sleeping sickness cases. Three *T*. *b*. *rhodesiense* infected animals being traded in Ochero market in Kaberamaido originated from Amolatar district, which had reported only a single reported human case.

A total of 67.9% of sampled cattle originated from within the market district itself while 18.9% were from districts to the south and 13.2% from districts to the north. Since samples were taken early in the market day, information regarding the future transport of infected animals was not available. Using the available permit movement records (from 8/10 of the sampled markets) and an estimated prevalence of 1.5% *T*. *b*. *rhodesiense*, an estimated 434 *T*. *b*. *rhodesiense* positive cattle (out of 28,949) will have been traded through these 8 markets to pathogen-free districts between mid-2006 to mid-2008.

### Activities of cattle restocking organisations

A total of 18 different organisations (3 governmental and 15 non-governmental) were identified by DVOs in seven districts in the Acholi and West Nile sub-regions as being involved in cattle restocking. Five of these districts had reported *T*. *b*. *gambiense* cases. Organisations ranged from large donor-sponsored governmental programmes such as the National Agricultural Advisory Services (NAADS) and the Northern Uganda Social Action Fund (NUSAF) to faith-based organisations such as the Lutheran World Federation and World Vision to international NGOs such as Oxfam and Heifer International. From record books and project reports, the three governmental organisations funded largely by international donors were responsible for the majority of restocking efforts, moving a reported 53,607 cattle between late-2006 and late-2008. The 15 NGOs were responsible for a reported 4,250 cattle moved during the same period (total of 57,857 cattle). In addition, the seven district DVOs considered that between 25% to 50% of cattle in their respective districts were imported by private traders and farmers and furthermore that this proportion had been progressively increasing from 2006 to 2008 (14,464 to 28,929 extrapolating from the 57,857 cattle reported by restocking organisations). From these records and observations, an estimated 72,321 to 86,785 cattle were imported into seven *T*. *b*. *rhodesiense*-free districts (Amuru, Gulu, Kitgum, Pader, Adjumani, Arua and Nebbi) between 2006 and 2008.

Organisations engaged in restocking activities concentrated mainly on the supply of cattle for draught with a few focussing on milk production and breeding schemes and this influenced the sourcing of cattle. Ankole, Frisian and Zebu cattle breeds are each associated with specific farming roles and sourced from different regions of Uganda. Although cattle were obtained from across a wide geographic range, including many districts in the central, southern, eastern and northern regions, 13 organisations (including the 3 governmental organisations) only sourced and acquired Zebu breeds (the remainder acquiring a mixture of Ankole, Frisian and Zebu). Zebu cattle are hardy draught animals that are regarded as less susceptible to indigenous cattle diseases and are found almost exclusively in the southeast of Uganda (endemic for *T*. *b*. *rhodesiense*). The higher milk yielding Ankole and Friesian breeds and crosses (generally considered more disease-prone) are predominantly located in the west and southwest of the country. As a result, the majority of cattle sourced by restocking organisations were acquired from districts in which *T*. *b*. *rhodesiense* is also endemic.

All three governmental organisations claimed to employ trained veterinarians to undertake cattle procurement and they purported to purchase at large district markets. In contrast, all bar one NGO (who claimed to use a qualified animal health assistant), reported using private contractors without any formal veterinary training to purchase, inspect and transport cattle. Veterinary inspections and treatments were left to the discretion (and expense) of private contractors, who were deemed liable if an animal died within a specified period of time after purchase. Most interviewees were unsure whether cattle were acquired from formal livestock markets or directly from villages.

Interviews related to Rhodesian sleeping sickness indicated that half of respondents (9/18) from restocking organisations possessed some basic knowledge of the disease (identifying the tsetse vector and naming the south-eastern sub-region as the main endemic foci in Uganda). Only 39% of respondents (7/18) were able to identify Rhodesian sleeping sickness as a zoonosis. When asked how to prevent sleeping sickness, respondents cited tsetse trapping (11/18), epidemiology (5/18), habitat destruction (6/18) and, to a lesser extent, vaccination and quarantine as possible solutions. No respondent identified trypanocide treatment of cattle as a prevention strategy and none were sure if the animals they were moving had been treated after being sold.

### Trypanocidal treatment at the 10 livestock markets

Direct observations and interviews at market sites revealed a number of significant constraints to trypanocidal treatment relating to sales practices, market infrastructure, animal health inspection procedures and the availability of veterinary drugs. A complete market perimeter fence important in limiting non-compliance with market regulations was observed in only 3 sites. Only one market complied with the government procedure of inspecting livestock prior to admittance to the market site while nearly half conducted animal health spot checks as animals were exiting the market. The remainder did not exhibit any form of animal health inspection procedure. A maintained and working market crush was observed in only 2 markets. Eight out of ten of the markets had some form of veterinary drug shop or itinerant drug seller in the vicinity that stocked trypanocidal drugs.

During 25 market day visits, regular trypanocidal treatments were only observed at 3 markets on 5 different occasions. However even at these markets, some animal owners or traders were observed to have avoided health inspections and left the market site without consulting the market veterinarian (often seated in one corner of the market). Sporadic treatment was also observed at several other markets. Although movement permits are in theory issued free of charge, most veterinarians charged a nominal fee of between 1,000 to 3,000 UgSH for a permit and inspection (approximately $0.50 to 1.50 USD). Any treatment involved additional costs. Injectable treatments were observed to be mostly with antibiotics and most farmers interviewed were not able to provide a rational for these treatments apart from ‘to help with diseases’. Interview and observational data showed that government restocking programmes (who procured cattle using private veterinarians) did often treat all cattle regardless of health-status with trypanocides obtained outside of the market area; private traders as well as operators used by NGO restocking organisations tended to avoid any health inspections unless the animal was notably ill. The veterinarians working for the governmental organisations that were interviewed clearly understood the importance of trypanocidal treatment at point of sale or movement. Over the course of the period 2006 to 2008, activities by restocking organisations had reduced, so that by the end of 2008 most trade was by private traders. No interviewed farmer or trader knew of the importance for trypanocidal treatment in preventing the spread of sleeping sickness. Since *T*. *b*. *rhodesiense* is asymptomatic in cattle, traders and contractors (whose main interest was in maximizing their profit margins) considered paying for treatment for a disease that an animal was not perceived to be suffering from as unnecessary. Animals destined for slaughter should not receive trypanocidal treatment 21 days prior to consumption and a number of interviewees claimed to deceive the veterinary staff in markets where treatment was enforced, to avoid the need for payments.

Most of the 20 interviewed DVOs from the northern and eastern regions were sceptical that trypanocidal treatments were undertaken regularly at markets within their districts (even by the government restocking schemes) and emphasised the high number of animals being traded outside of the market system. Many market veterinarians were confused about whether trypanocidal treatment was mandatory or was only ‘a suggestion’ from the central government. This was at least partially driven by the fact that (as DVOs pointed out) the policy was only a directive from the Ministry of Agriculture (MoA) and it lacked a legal basis for enforcement. Treatment was therefore done at the expense and willingness of the cattle buyer and only occasionally enforced by some dedicated veterinary staff. Veterinarians were observed to use their own drugs for treatments, clearly benefiting financially by enforcing what was viewed as an ambiguously defined policy.

Assuming that the three government restocking schemes consistently treated cattle with trypanocides while NGOs and private traders did not, out of 72,321 to 86,785 cattle imported into seven *T*. *b*. *rhodesiense*-free districts in the Acholi and West Nile sub-regions between 2006 and 2008 (based on the restocking organisation estimates), at least 18,714 to 33,179 cattle would not have received trypanocidal treatment prior to being moved. Most of the Zebu animal trade would have originated from endemic Rhodesian sleeping sickness areas. Due to poor records, illegal trading and the more realistic assumption (advanced by most DVOs) that not all government organisations consistently adhered to treatment, this is likely to be an underestimate. Assuming that all imported cattle came from endemic market sites and extrapolating from the prevalence data (1.5%) obtained from the 10 sample markets, between at least 1085 to 1302 infected cattle would have been purchased from endemic markets and moved into the pathogen-free northern districts. The number of infected animals not treated with trypanocides would have ranged from between 281 to 498 (assuming all government organisations consistently adhered to treatment) to 1085 to 1302 (assuming no animal received treatment). These would have been imported to different districts and different areas within single districts.

## Discussion

With the end to military conflict in northern Uganda, efforts to restock post-conflict districts with cattle have pushed Rhodesian sleeping sickness further towards northern districts endemic for *T*. *b*. *gambiense*. Overlap between the two forms of sleeping sickness would severely complicate disease diagnosis, treatment and control in areas with some of the highest poverty rates in Uganda and already fragile and under-resourced health systems. Much of the northwest is tsetse-infested and sustained movement of *T*. *b*. *rhodesiense* positive cattle could drive a large-scale epidemic if the parasite were to establish itself in the area. Porous borders, human migration and cattle trading from the West Nile and Acholi sub-regions could also move infected cattle into South Sudan and the Democratic Republic of Congo where some of the most significant *T*. *b*. *gambiense* foci are located [18]. The scale of cattle movements, its repercussions and the various drivers that may increase or decrease the likelihood of an emerging epidemic have not been fully appreciated.

Here we investigated the likelihood of parasite introduction to the post-conflict northern region by examining the extent and direction of cattle trading from *T*. *b*. *rhodesiense* endemic districts between 2006 and 2008, the prevalence of the parasite in cattle traded from these markets as well as the level of adherence to trypanocidal treatment at market sites and the practices of cattle restocking organisations and private traders. The study estimated cattle movements based on three different sources of data: movement permit records from 8 livestock markets in *T*. *b*. *rhodesiense* endemic areas (mid-2006 – mid-2008), district level records from Lira district (September 2007-May 2008), and figures obtained from 18 restocking organisations (late-2006 to late-2008) together with interview data from DVOs. These different data sets have shown that a significant amount of cattle (an estimated 28,949 cattle from the 8 markets; 11,199 from Lira district; and between 72,321 and 86,785 estimated cattle from the restocking organisations’ data) were moved from *T*. *b*. *rhodesiense* endemic districts to pathogen-free areas of northern Uganda. Much of this was to *T*. *b gambiense* endemic areas. The records based on official movement permit records from only 8 markets compared to the total 47 identified in the 13 *T*. *b*. *rhodesiense* districts underestimated the full extent of cattle movements, especially when compared to the figures from Lira district. Some restocking organisations were also likely not consulted and others may have underrepresented the scale of their activities due to what were observed to be poor record keeping. During the post-conflict period many cattle traders were known to source their cattle directly from villages, seek a movement permit from the DVOs office directly, evade movement permits altogether at markets (and transport cattle illegally), or obtain a movement permit for only a few of the animals they intended to transport. Accurately estimating cattle movements in the immediate post-conflict period in Uganda is difficult since records were often unavailable, incomplete or of questionable accuracy.

The three large government organisations responsible for most of the formal restocking used trained veterinary staff that adhered to guidance and provided trypanocidal treatments. However many DVOs expressed reservations about the consistency of these treatments and it is likely that not all of these animals were treated; the exact proportion of treated to untreated cattle is not possible to quantify. Cattle were also imported by NGO contractors and private cattle traders and farmers who were largely unaware of the importance of trypanocidal treatment and only rarely treated animals sold at markets in order to reduce costs and maximise profits. This private trade was found to be increasing while the activity of restocking organisations had reduced significantly by 2009. Direct observations at the 10 livestock markets showed that a lack of basic market infrastructure and willingness of most market veterinary staff to enforce treatment together with the need for payment, lack of awareness and unregulated livestock trading conducted outside of markets sites contributed to most cattle not being treated with trypanocides. Market staff and DVOs expressed confusion about the nature of the trypanocidal treatment policy and felt powerless to enforce a directive from the MoA that they felt lacked a legal basis.

Based on the *T*. *b*. *rhodesiense* prevalence of 1.5% found in cattle being sold across 10 livestock markets in endemic districts of Uganda, we have also estimated the number of infected animals likely moved to disease-free northern districts as between at least 1085 to 1302 cattle. The number of infected animals not treated with trypanocides would have ranged from between 281 to 1302. Using the same prevalence data, an additional estimate was provided from the available movement permit records from the 8/10 markets which showed that 434 *T*. *b*. *rhodesiense* infected cattle (out of an estimated 28,949) would have been purchased from these 8 markets and moved to pathogen-free districts between mid-2006 to mid-2008; most of which was likely not treated prior to being imported. Research from Soroti district has shown that the importation of 100 cattle from an endemic region over a four-year period was sufficient to establish the parasite in a naïve area [32,33].

Livestock movement regulations are a recognized method of epizootic and zoonotic disease control with international standards provided by the World Organisation for Animal Health (OIE) [44]. While Uganda’s Animal Diseases Act (1918) clearly states that all animals being moved into a new district require the written permission and inspection of the local veterinary officer, this policy has not been updated since the colonial era and animal *trypanosomiasis* is not considered a notifiable disease [20,34]. Preventing the spread of an infectious human pathogen into a disease-free area is recognised as cost-effective and morally advisable if human lives can be saved. Some scholars have commented on the politics of disease control prioritization and the various interest groups and policy narratives that shape them [45]. For economically significant trans-boundary diseases such as Foot and Mouth Disease (FMD) and others in Africa, the international community and national governments are more willing to invest in and implement strict livestock movement regulations. While mandatory trypanocidal treatment has not been consistently employed, a number of enforced quarantines for FMD have been implemented in eastern Uganda since 2009. This resulted in substantial economic hardship for many poor farmers as FMD quarantines may last for several months. Additionally, one of the last remaining endemic areas for rinderpest in Africa was the remote north-eastern Karamoja sub-region in Uganda responsible for the cattle raids on the rest of the northern region in the late-1980s. Despite dangerous conditions, in the 1990s concerted capacity building, the development of the thermostable rinderpest vaccine and the incorporation of community-based animal health workers in vaccination campaigns successfully eradicated the disease [46]. These successful mandatory ‘top down approaches’ were driven and financially supported by the ‘pro-eradication’ policy narratives shaped by international organisations [47]. With appropriate support, trypanocidal treatment at livestock markets should also be promoted and enforced in these post-conflict areas.

Affecting mostly poor and marginalised communities, many endemic zoonotic diseases such as Rhodesian sleeping sickness have been relatively neglected by international organisations and national governments [47]. History shows that the movement of *T*. *b*. *rhodesiense* is costly in both economic and public health terms. For example, the northern movement of the parasite to Soroti district in 1998 and then Dokolo, Kaberamaido and Lira districts in 2004 has contributed to an ongoing low-grade epidemic with over 1,300 reported, and many more unreported, cases. While the disease is costly to patients and their families [2-4], a significant amount of donor and government funding has been spent in these areas, including establishing treatment centres, sensitising the public, conducting intermittent active surveillance, deploying tsetse traps and mass treating cattle with trypanocides and acaricides. While mass treatment of over 250,000 cattle by the Stamp Out Sleeping Sickness (SOS) campaign between 2006 and 2010 significantly reduced the prevalence and northern range of *T*. *b*. *rhodesiense* in these districts, continued ingress of infected cattle from the south-east will continue to fuel outbreaks and pose a risk of disease overlap [38]. Trypanocidal treatment of cattle sold at markets, or at point of movement to new districts offers a cost-effective risk mitigation strategy for zoonotic sleeping sickness and animal trypanosomiasis.

With the continued economic recovery of northern Uganda and continued demand for Zebu draft oxen, cattle movements will continue into the immediate future. Preventing infected cattle (especially Zebu cattle) from introducing human pathogens into naïve areas is paramount. Moreover animals are being moved by private traders, and individual farmers, who do not treat these newly purchased cattle and who actively avoid trypanocidal treatment at market sites. While increased cattle numbers will help to alleviate poverty in the area, importation of cattle poses a significant risk to the continued spread of Rhodesian sleeping sickness into new areas. This will ultimately result in the spatial overlap of the two forms of human trypanosomiasis in Uganda (and if pushed further north, into South Sudan and/or the DRC). Specific environmental drivers (for instance, biophysical features that affect tsetse populations) that may increase or decrease the likelihood of an epidemic in the northern region are relatively poorly understood. A study in the West Nile sub-region found no human-infective trypanosomes in a variety of domestic animals [48] but most of the samples were obtained before the end of conflict in the northern region in 2006. In 2012, Rhodesian sleeping sickness cases were reported to the west of the established foci in Lira, in Kole district which suggests that *T*. *b*. *rhodesiense* continues to spread slowly northwards. Formal policy adoption for the mandatory treatment of cattle at point of sale in all endemic districts is urgently needed to prevent further spread. Policy should be implemented alongside a strengthening of market infrastructure and the capacity of market veterinarians to enforce treatment. Since Rhodesian sleeping sickness is a public health issue, the medical sector, government and/or international bodies should consider drug subsidisation in accordance with the One Health approach. Alternatively, switching the payment structure to have the seller pay for treatment instead of the buyer (for instance, by implementing a market entrance fee) could also help with compliance.

## Conclusion

Here we have explored the likelihood of the introduction of *T*. *b*. *rhodesiense* into post-conflict areas of northern Uganda between 2006 and 2008. As demand for draft oxen (predominately Zebu cattle) and private cattle trading continues to bring infected animals from endemic to pathogen-free areas of northern Uganda, treatment of cattle at point of sale at markets is an essential measure preventing continued expansion of the *T*. *b*. *rhodesiense* focus and risk of human disease overlap with the Gambian sleeping sickness focus. Although implementation of effective zoonotic disease control in resource-poor areas may appear challenging, the costs of neglecting to prevent the future spread of *T*. *b*. *rhodesiense* will be much higher both for subsistence-level farmers and governments and international donors.

## Competing interests

The authors declare they have no competing interests.

## Authors’ contributions

Conceived and designed the experiment: RS, CW, SCW, KP. Performed the experiment: RS. Analyzed the data: RS, KB. Contributed reagents/materials/analysis tools: RS, KB, KP. Wrote the paper: RS, KB, SCW. All authors read and approved the final version of the manuscript.
